# Chemical vapor deposition of germanium-rich CrGe*_x_* nanowires

**DOI:** 10.3762/bjnano.12.100

**Published:** 2021-12-07

**Authors:** Vladislav Dřínek, Stanislav Tiagulskyi, Roman Yatskiv, Jan Grym, Radek Fajgar, Věra Jandová, Martin Koštejn, Jaroslav Kupčík

**Affiliations:** 1 Institute of Chemical Process Fundamentals, Czech Academy of Sciences, Rozvojová 2/135, 165 02 Prague 6, Czech Republic; 2 Institute of Photonics and Electronics, Czech Academy of Sciences, Chaberská 1014/57, 182 51 Prague 8, Czech Republic

**Keywords:** chemical vapor deposition, chromium germanide, nanostructured materials, nanowire, resistivity

## Abstract

Chemical vapor deposition was applied to synthetize nanostructured deposits containing several sorts of nanoobjects (i.e., nanoballs, irregular particles, and nanowires). Analytical techniques, that is, high-resolution transmission electron microscopy, scanning electron microscopy, electron dispersive X-ray analysis, selected area electron diffraction, and X-ray photoelectron spectroscopy, showed that unlike nanoballs and particles composed of crystalline germanium, the layer was made of chromium germanide CrGe*_x_*. The nanowires possessed a complex structure, namely a thin crystalline germanium core and amorphous CrGe*_x_* coating. The composition of the nanowire coating was [Cr]/[Ge] = 1:(6–7). The resistance of the nanowire–deposit system was estimated to be 2.7 kΩ·cm using an unique vacuum contacting system.

## Introduction

Metal silicides and germanides belong to an extensively studied group of materials offering a wide variety of properties to meet various requirements in battery, optical, and electronic applications, as well as catalysis. Moreover, their compatibility with silicon technology is advantageous. The rising need for advanced technologies in IT, aerospace, chemical, and power supply industries has accelerated the search for novel materials that could be applied along with traditional materials or even replace them due to their better physicochemical properties. However, while research on plenty of metal silicides is already underway, we are only at the beginning with germanides. For example, CuSi*_x_* has been studied extensively for some time. There is even an important application of copper silicide in the chemical industry, the so-called “direct (Müller–Rochow) process” in which chloromethane and silicon are converted in various chlorosilanes [1]. In contrast, only few papers about CuGe*_x_* have been published, in which a few possible applications were mentioned and tested. Another example is chromium silicide/germanide. CrSi*_x_* has been described in tens of publications and at least some properties of it are known and classified. However, only few CrGe*_x_* publications have been published so far. Although silicon and germanium as representatives of group-IV elements possess similar physicochemical characteristics, their thermodynamic properties hint at quantitatively and qualitatively different behavior of their metal alloys. Therefore, germanides as counterparts of silicides have been gradually becoming a topic of current research.

Thermodynamic properties of chromium germanide CrGe*_x_*, that is, the phase diagram [2] and Gibb’s energies [3], were determined. In other works, the crystallographic phases Cr_3_Ge, Cr_5_Ge_3_, Cr_11_Ge_8_, CrGe, and Cr_11_Ge_19_ were synthetized using chemical vapor transport [4]. Also, Cr_11_Ge_19_ in the form of large single crystals was obtained using a two-zone vertical gradient freeze furnace. Cr_11_Ge_19_ belongs to the large family of compounds exhibiting a Nowotny chimney ladder crystal structure. Such materials have mostly significant thermoelectric properties [5]. CrGe superlattices in CrGe/FeGe and CrGe/Mn/Ge/FeGe systems were fabricated for advanced materials with tunable skyrmions [6]. In another theoretical work, a Cr@Ge_10_ nanocluster was shown to be a candidate for a transition metal-doped magnetic superatom [7], which behaves as if it was one atom. Unique magnetic properties have been found in diluted magnetic semiconductor (DMS) alloys [8]. Silicon and/or germanium are a reasonable choice for DMS alloys with chromium for the best compatibility with silicon-based industry. However, precipitation of transition metals is the main obstacle; but in low-dimensional semiconductors the precipitation is significantly reduced. Therefore, CrGe nanowires (NWs) were prepared to study their magnetic defects and their interactions with charge carriers. Antiferromagnetic clusters in CrGe NWs were investigated using electron spin resonance. Spin–orbit interaction between charge carriers and magnetic defects were studied [9]. Cr/Ge nanotowers as a dilute magnetic semiconductor were prepared, too. Magnetic properties were measured and the growth mechanism was discussed [10]. The formation of Cr/Ge nanoparticles during the epitaxial growth of Cr/Ge films and their magnetic properties were studied to understand the ferromagnetic semiconductor behavior [11].

In this work, we made an effort to prepare a Cr/Ge deposit in a nanostructured form. Using chemical vapor deposition (CVD), we succeeded to synthesize deposits containing CrGe*_x_* NWs. Their structure was elucidated and measurements of individual NWs were carried out to determine their electrical resistivity.

## Results and Discussion

**Figure 1:**
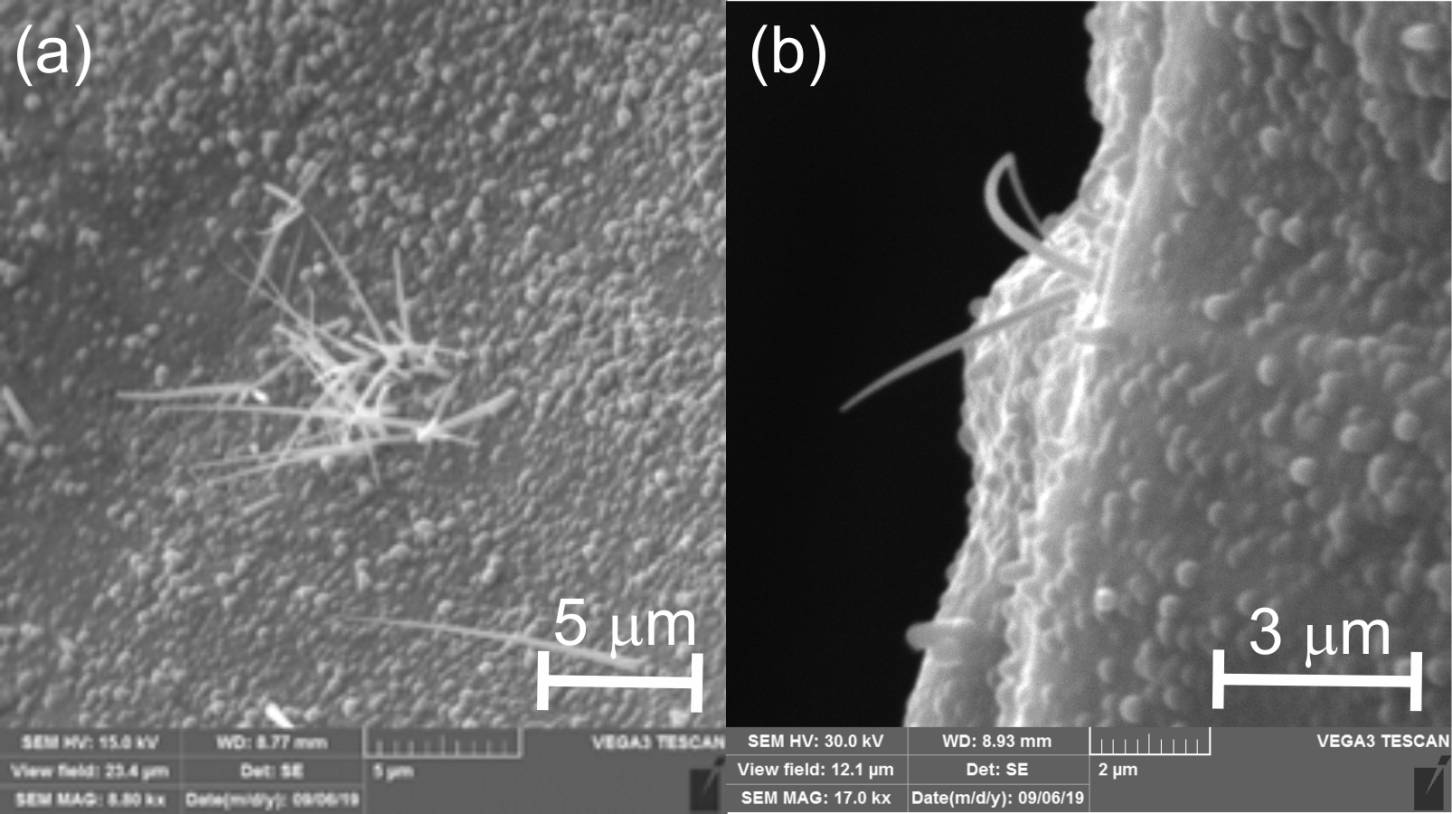
(a) SEM image of a Cr/Ge deposit with nanowires (b) growing in a tapering manner.

**Figure 2:**
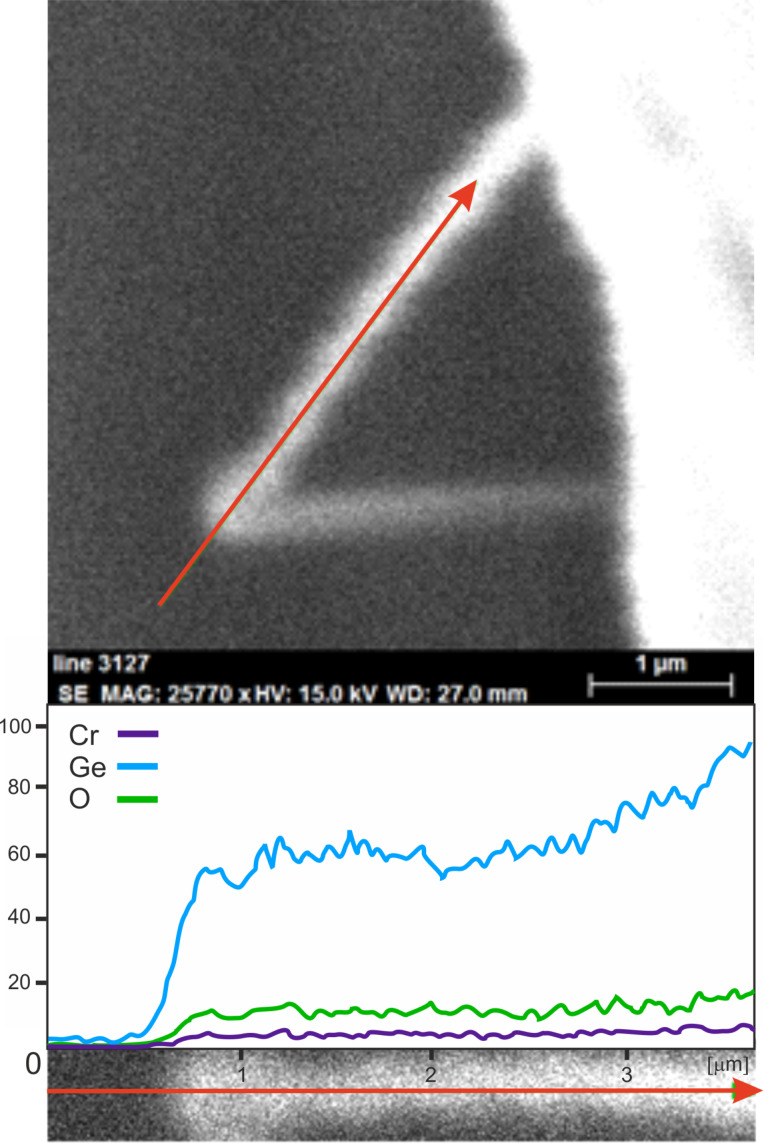
Linear EDX analysis along a single nanowire.

Molybdenum substrates that were placed at the very beginning of the second part of the furnace were covered by a deposit (Figure 1a) containing chromium, germanium, and oxygen (Supporting Information File 1, Figure S1). The [Cr]/[Ge] atomic ratio was in the range of 1:(8–12). From a granular-like surface, nanowires grew in a tapering manner (Figure 1b and Supporting Information File 1, Figure S2). Linear EDX analysis (Figure 2) and elemental mapping (Supporting Information File 1, Figure S3) of a single nanowire showed that it was composed of Cr, Ge, O (from an oxidized surface) and C (a standard impurity in EDX instruments).

**Figure 3:**
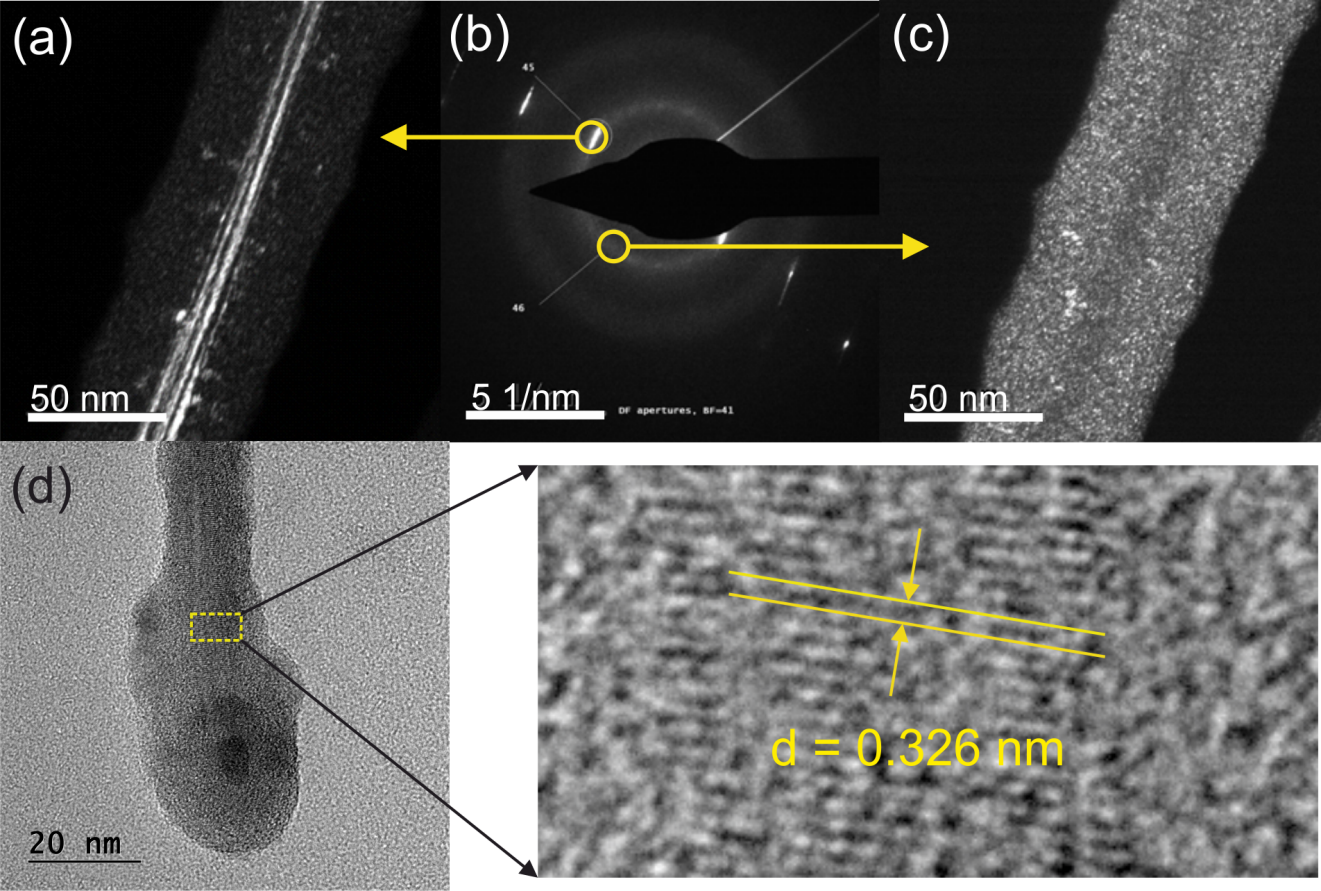
(a, c) Dark-field HRTEM images, (b) SAED of a nanowire piece, and (d) HRTEM image of a NW top piece with magnified crystalline core and a determined *d*-spacing of 0.326 nm. The dark-field image corresponds to an indicated diffraction spot in the SAED image. Diffuse circles and individual points indicate the amorphous coating and crystalline germanium, respectively.

HRTEM analysis showed three types of synthetized nanoobjects: tapered NWs (Supporting Information File 1, Figure S4a) and objects of irregular (Supporting Information File 1, Figure S4b) and globular shape (nanoballs, Supporting Information File 1, Figure S4c). The nanoballs are composed of crystalline cubic germanium that is covered with a thin layer of GeO*_x_* (Supporting Information File 1, Figure S5). Irregularly shaped particles are formed from several phases of germanium, that is, cubic, hexagonal, and amorphous Ge (Supporting Information File 1, Figure S6). A close look at the nanowire using SAED, dark-field HRTEM, and EDX analysis showed that it consisted of a crystalline germanium core sheathed with an amorphous Cr/Ge coating (Figure 3 and Supporting Information File 1, Figure S7) resembling SiNWs with similar structure [12]. The determined *d*-spacing of 0.326 nm fits precisely with the database PDF (00-004-0545) for cubic germanium (*d*_111_ = 0.3266 nm). The atomic elemental ratio [Cr]/[Ge] in the coating was in the range of 1:(6–7) (Supporting Information File 1, Figure S8).

**Figure 4:**
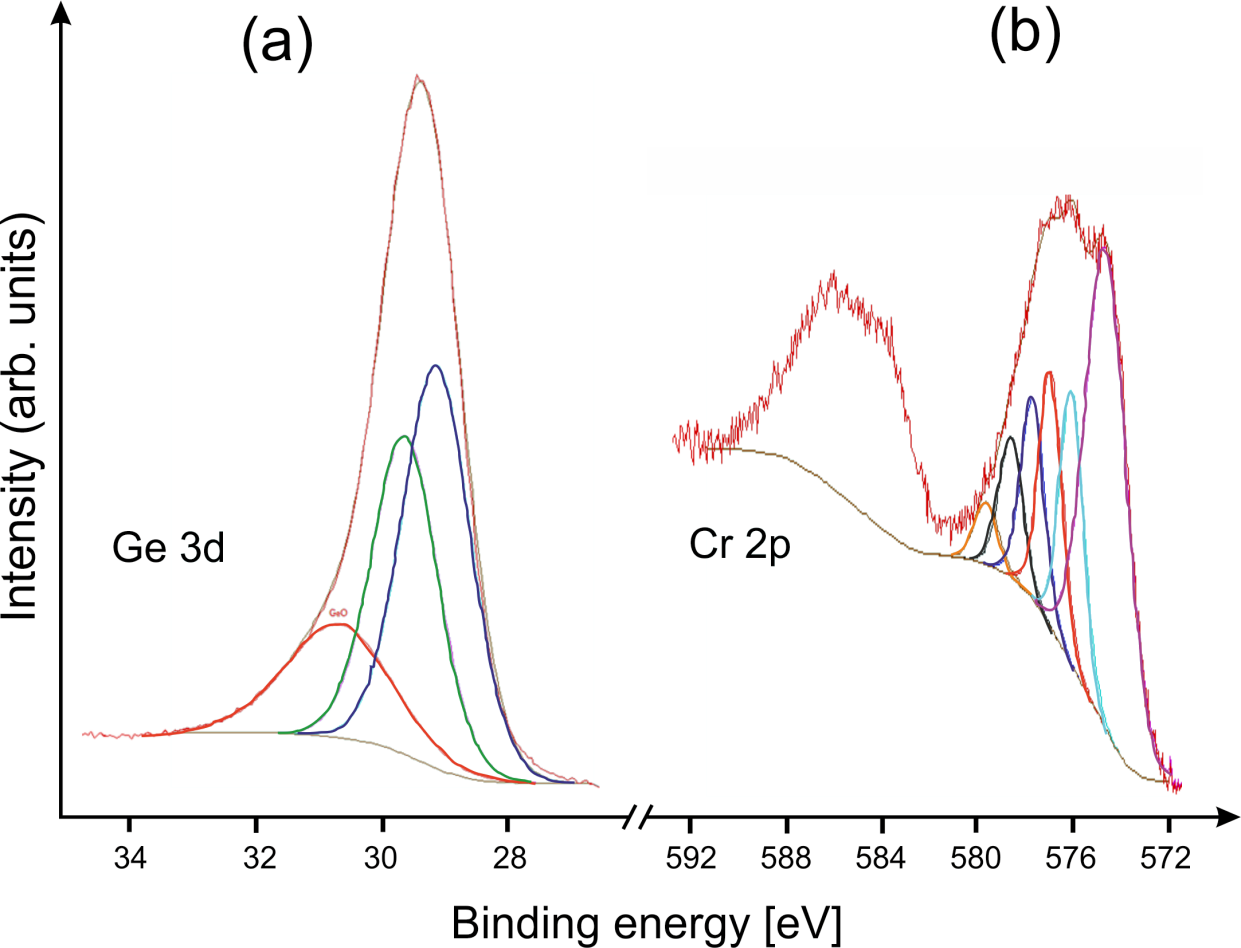
XP spectra of (a) the Ge 3d and (b) the Cr 2p_3/2_ region after 90 s of Ar^+^ ion sputtering.

XP spectra showed the presence of Ge, Cr, and O. In order to reduce surface contamination and oxidized species, Ar^+^ ion sputtering was used. The initial atomic elemental ratio was [Cr]/[Ge]/[O] = 1:7.33:11.61; after 90 s of Ar^+^ ion sputtering the ratio was [Cr]/[Ge]/[O] = 1:4.41:2.43. The presence of oxygen indicated oxidation after the experiment. The [Cr]/[Ge] atomic elemental ratio of 1:4.41 was significantly higher than the one obtained by EDX, namely 1:(8–12), which could be explained by chromium enrichment in the superficial surface layer of the deposit after its exposure to air. Elemental analysis obtained from the coating of individual nanowires showed this ratio to be in the range of 1:(8–10), which was in accordance with EDX measurements of a large area of the deposit. Ge 3d components (Figure 4a) showed elemental Ge/metal germanide (29.5, 29.0 eV) and a small amount of GeO (30.6 eV) formed by oxidation. The chromium Cr 2p_3/2_ peak was deconvoluted into a component at 574.4 eV and five multiplet peaks at higher binding energies associated with elemental Cr/germanide (CrGe*_x_*) and multiplet splitting of the Cr(III) oxide species [13], respectively (Figure 4b). We were not able to separate elemental Cr and CrGe*_x_* subpeaks as the XPS shift of a metal (chromium)/germanium in metal (chromium) germanides relative to the pristine metal (chromium)/germanium is only several tens of electronvolts [14–15].

Germane GeH_4_ decomposes thermally according to [16]:


[1]

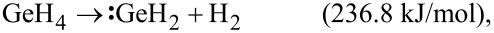



whereas the next reaction is not favored [16]:


[2]

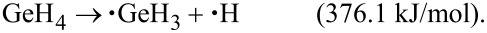



Paralelly, chromium(III) acetylacetonate decomposes via [17]:


[3] $${\text{Cr(acac)}}_{3}\to {\text{Cr(acac)}}_{3-x}\text{ (}x\text{ = 2,3).}$$

The products of the above reactions undergo subsequent reactions, mostly reduction reactions resulting in the formation bulk Ge (in excess GeH_4_) and CrGe*_x_*. Ge atoms tends to create islands and then nanoballs. We do not fully exclude that some oxygen in GeO*_x_* on the nanoball surfaces originate from acetylacetonate residues. The nanoballs serve as initiation spots from which Ge NWs grow via a self-catalyzed mechanism. The Nanowires grow from the top as one can see a small nanodroplet on the top in Figure 3d. It means that growth follows a vapor–liquid–solid (VLS) mechanism [18], although the material of droplet and NW is the same. Bear in mind that the melting point of nanodroplets may be reduced by hundreds of kelvins [19]. Tapering of the CrGe*_x_* coating is observed during the whole experiment as Cr and Ge atoms migrate from the NW bottom and/or are transferred directly from the gas phase [20].

Several attempts were made to transfer single NWs onto contact lithographic pads (Supporting Information File 1, Figure S9) to measure their conductivity. The NWs, however, turned out to be fragile and were destroyed when an attempt was made to cut them from the tungsten tip using a focused ion beam (FIB). Therefore, a method to directly contact an as-grown single NW was developed. This method allowed us to measure the conductivity between the molybdenum substrate and the point of contact of the tungsten tip with the NW. To limit the contact resistance between the tungsten tip and the NW, the tip was soldered to the NW with a carbon–platinum composite using focused electron beam-induced deposition (FEBID) (Supporting Information File 1, Figure S10).

**Figure 5:**
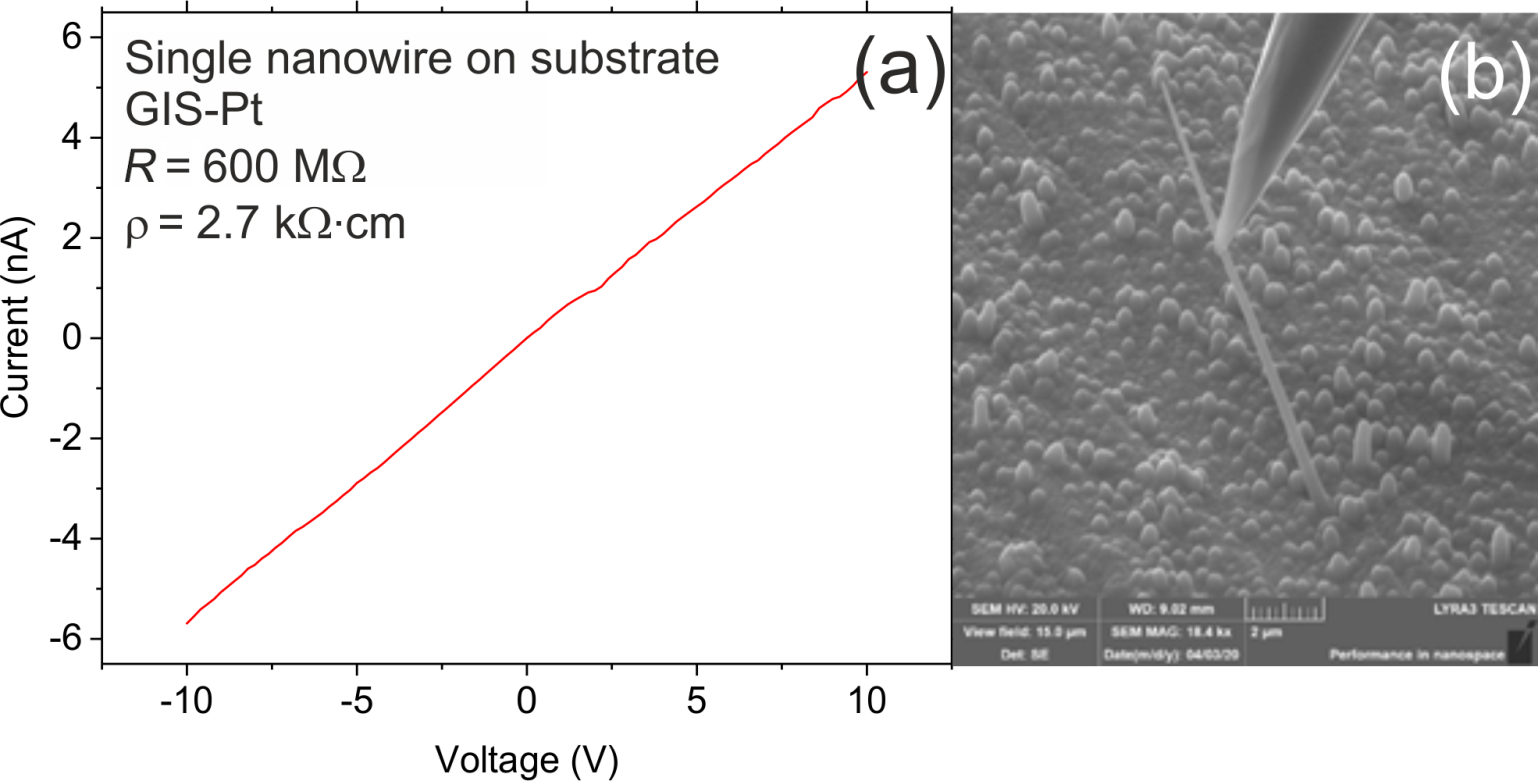
(a) *I*–*V* characteristics of the nanowire–deposit system; (b) SEM image of a contacted single CrGe*_x_* nanowire before the resistivity measurement.

The resistivity of the nanowire–deposit system was estimated to be 2.7 kΩ·cm (Figure 5). This value is significantly higher than the previously reported resistivity for nominally undoped Ge nanowires or intrinsic bulk germanium [21]. One of the possible explanations is that Cr can form deep levels within the Ge bandgap [22]. These levels act as recombination centers and increase the resistivity. However, a precise determination of the influence of Cr incorporation on the resistivity of the nanowire–deposit system would require a more detailed investigation. Another explanation is based on the growth mechanism of nanowires. CrGe*_x_* nanowires grow via a self-catalyzed mechanism (without any external seed). A generally accepted idea for self-catalyzed (non-catalyzed) nanowire growth [23] is that the growth of any nanowire has to be initiated first. Germanium crystalline nanoballs formed on the surface serve as initiation spots from which the nanowires start to grow. It is clearly evident from the images that the nanowire bottom was originally a nanoball with a mean diameter of 400–500 nm (Supporting Information File 1, Figure S11). Nanoballs have a thin oxidized surface shell (approx. 5 nm), as mentioned above (Supporting Information File 1, Figure S7b). Those nanoballs form in a CrGe*_x_* surrounding. The oxidized surface shell of the nanoball serves as an additional resistivity component in the nanowire–deposit system.

## Conclusion

Using CVD, we synthetized deposits containing CrGe*_x_* NWs. The complex elemental composition was determined, the structure was elucidated, and resistivity measurements of individual NWs were performed and discussed. These measurements revealed the influence of the structure of the deposit on the NW growth.

Although Cr/Ge possesses some advantageous thermoelectric properties, it seems that, currently, its magnetic behavior is more promising. Therefore, more electric and magnetic studies are needed. Germanium-rich Cr/Ge materials are regarded as representatives of diluted magnetic semiconductors, which have been intensively studied for future magnetic, optomagnetic, electronic, and related devices. Preparation of Cr/Ge materials in the form of NWs opens space for tuning the magnetic properties in germanium-rich Cr/Ge nanostructures. Moreover, the complex structure of the prepared nanowires, unlike simple structures, enables further more extensive engineering of nanowire properties by specific technological steps (e.g., thermal annealing, etching, doping, and filling) in order to obtain, for example, catalytic nanowires with huge specific surface or hollow/filled nanoscale cables suitable for medicinal magnetic transport.

## Experimental

CrGe*_x_* deposits were synthetized using CVD. In a custom-made twin furnace (Supporting Information File 1, Figure S12), chromium(III) acetylacetonate powder (Sigma-Aldrich, 99.99%) was heated to 110 °C and evaporated in the first part of the furnace. The Cr(acac)_3_ vapor in a continuous flow (3 sccm) of germane (GeH_4_) at 250 Pa was transported to the second part of the furnace where pyrolysis took place at 500 °C over molybdenum substrates.

X-ray photoelectron spectrometry (XPS, Kratos ESCA 3400 with a base pressure below 5.0 × 10^−7^ Pa) was used with a polychromatic Mg X-ray source (Mg Kα, 1253.4 eV). A standard superficial surface sputtering with Ar^+^ ions was applied using 1 kV of acceleration voltage. The Shirley background was subtracted for all spectra.

The morphology of each sample was analyzed by a scanning electron microscopy (SEM) setup (TESCAN Vega 3 InduSEM) equipped with an electron dispersive X-ray spectrometer (EDX, Bruker XFlash Detector 5010) for detecting the elemental composition. A standard automatic instrumental background was applied for the estimation of elemental composition.

Individual nanoobjects were studied by transmission electron microscopy (TEM) performed on a Philips CM 120 (LaB_6_, 120 kV) equipped with a NanoMEGAS precession unit DigiStar, an Olympus SIS CCD camera Veleta (2048 × 2048 px), and a windowless detector Apollo XLTW for EDX measurements. Selected area electron diffraction (SAED) patterns were evaluated using the ProcessDiffraction software package. The samples were prepared on holey carbon-coated Au/Mo grids by brushing the grids against the substrate containing the deposit.

Electrical measurements of the NWs were carried out in the SEM apparatus (Tescan Lyra 3) equipped with the Ga^+^ focused ion beam (FIB), gas injection system (GIS), and nanomanipulator OmniProbe 400 (Oxford Instruments) with a tungsten tip. The nanomanipulator enabled a direct contact of single as-grown NWs. The current–voltage (*I*–*V*) characteristics were measured using a Keithley 237 source measurement unit with the bias applied to the tip, while the substrate was grounded.

## Supporting Information

File 1 Analysis of CrGe*_x_* nanowires, experimental contacting for *I*–*V* characteristics, and scheme of CVD apparatus.
