# Supplementary material for: Chemical vapor deposition of germanium-rich CrGex nanowires
Source: Beilstein J Nanotechnol. 2021 Dec 7;12:1365–71. doi: 10.3762/bjnano.12.100 (PMC8685558; doi:10.3762/bjnano.12.100)
Supplement: File 1 — Analysis of CrGex nanowires, experimental contacting for I–V characteristics, and scheme of CVD apparatus. [file Beilstein_J_Nanotechnol-12-1365-s001.pdf]

BEILSTEIN JOURNAL OF NANOTECHNOLOGY

Supporting Information

for

Chemical vapor deposition of germanium-rich $CrGe_x$ nanowires

Vladislav Dřínek, Stanislav Tiagulskyi, Roman Yatskiv, Jan Grym, Radek Fajgar, Věra Jandová, Martin Koštejn and Jaroslav Kupčík

Beilstein J. Nanotechnol. **2021,** *12,* 1365–1371. doi:10.3762/bjnano.12.100

Analysis of $CrGe_x$ nanowires, experimental contacting for *I–V* characteristics, and scheme of CVD apparatus

License and Terms: This is a supporting information file under the terms of the Creative Commons Attribution License (https://creativecommons.org/licenses/by/4.0). Please note that the reuse, redistribution and reproduction in particular requires that the author(s) and source are credited and that individual graphics may be subject to special legal provisions.
The license is subject to the *Beilstein Journal of Nanotechnology* terms and conditions: (https://www.beilstein-journals.org/bjnano/terms)

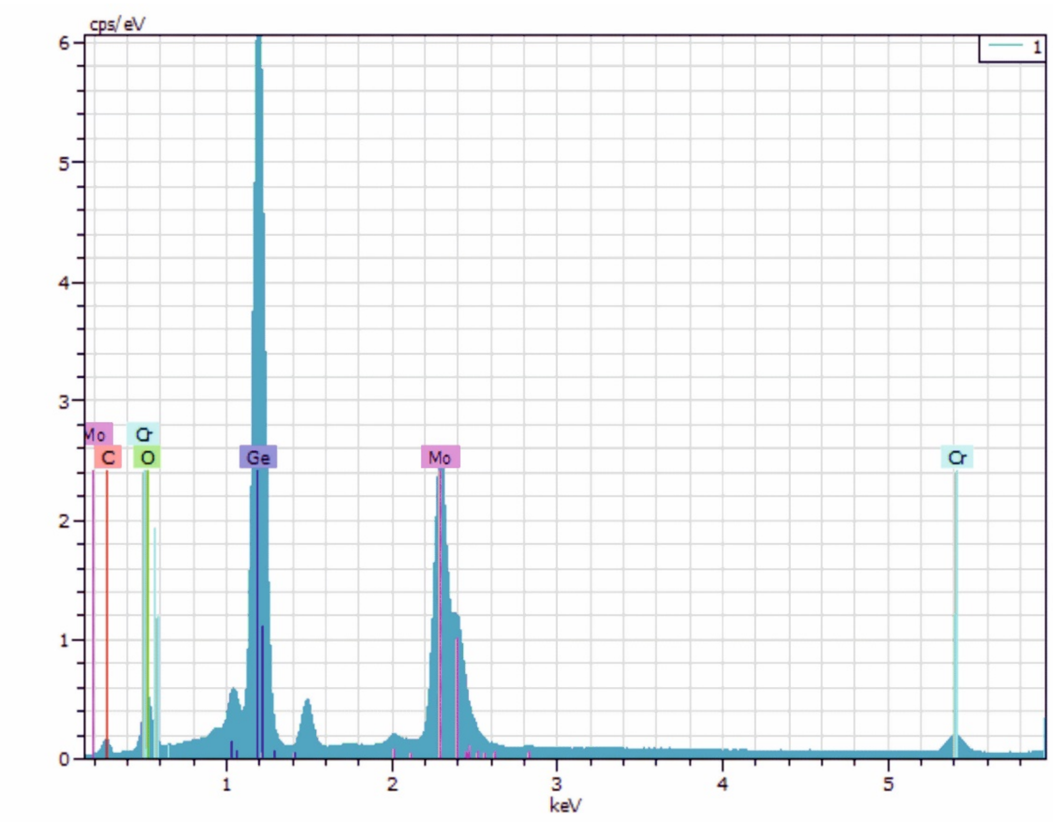

El	AN	Series	unn. C [wt.%]	norm. C [wt.%]	Atom. C [at.%]	Error (1 Sigma) [wt.%]
C	6	K-series	6.52	5.59	22.55	0.93
O	8	K-series	9.58	8.22	24.87	1.20
Cr	24	K-series	3.48	2.99	2.78	0.13
Ge	32	L-series	56.14	48.16	32.11	3.15
Mo	42	L-series	40.85	35.04	17.68	1.45
		Total:	116.59	100.00	100.00	

Figure S1: EDX analysis of a Cr/Ge deposit.

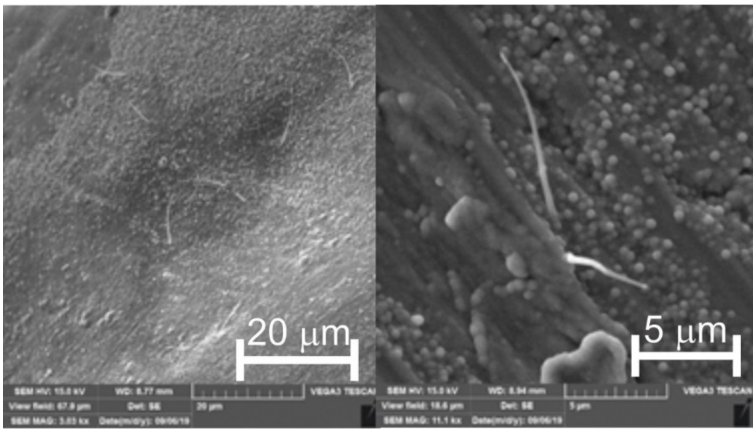

Figure S2: Tapered nanowires scarcely growing from a deposit.

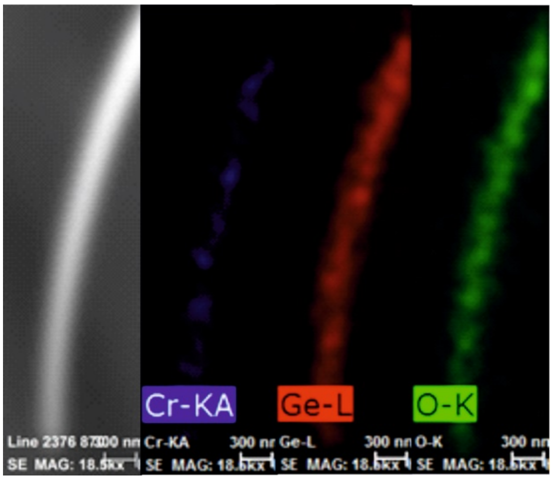

Figure S3: Elemental mapping of a single nanowire.

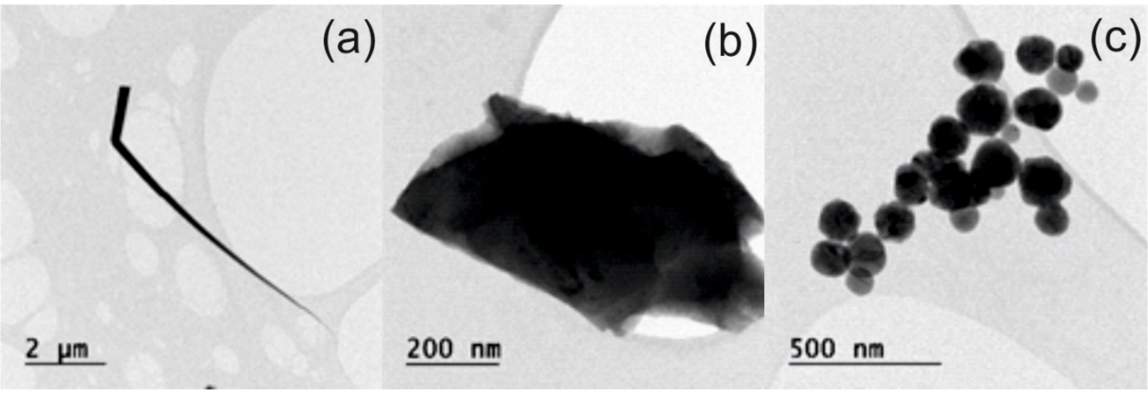

Figure S4: Nanoobjects observed during HRTEM analysis: (a) nanowires, (b) objects of an irregular shape, and (c) nanoballs.

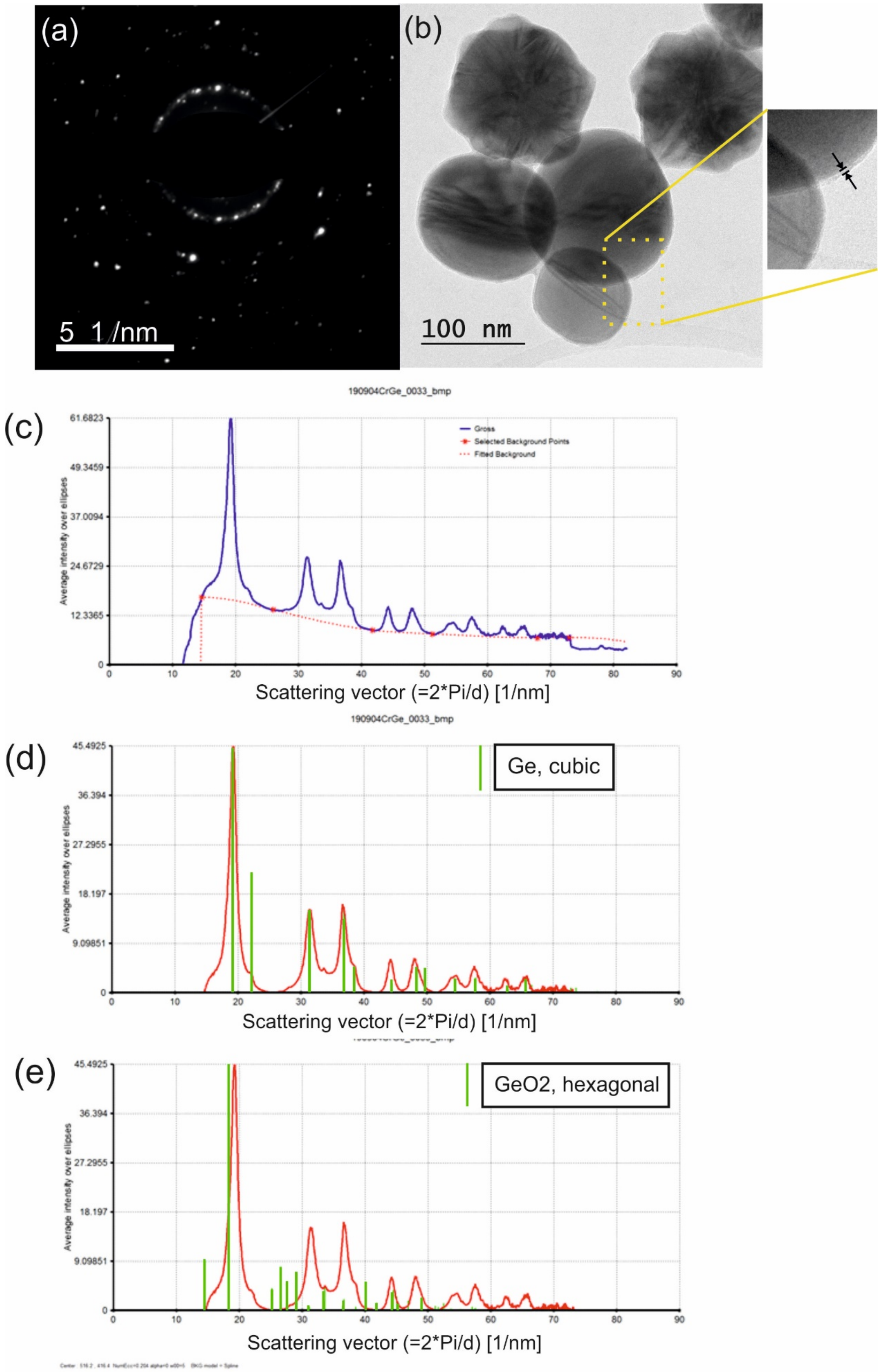

Figure S5: SAED analysis of nanoballs. (a) SAED image, (b) HRTEM photo with part of nanoballs magnified to make visible an oxidized layer (~5 nm) on the nanoball surface, (c) SAED image converted into an intensity diffractogram, before background substraction, and (d) Ge cubic (PDF 65-9209) and (e) GeO_2 hexagonal (PDF 85-1515) markers in diffractogram after background substraction.

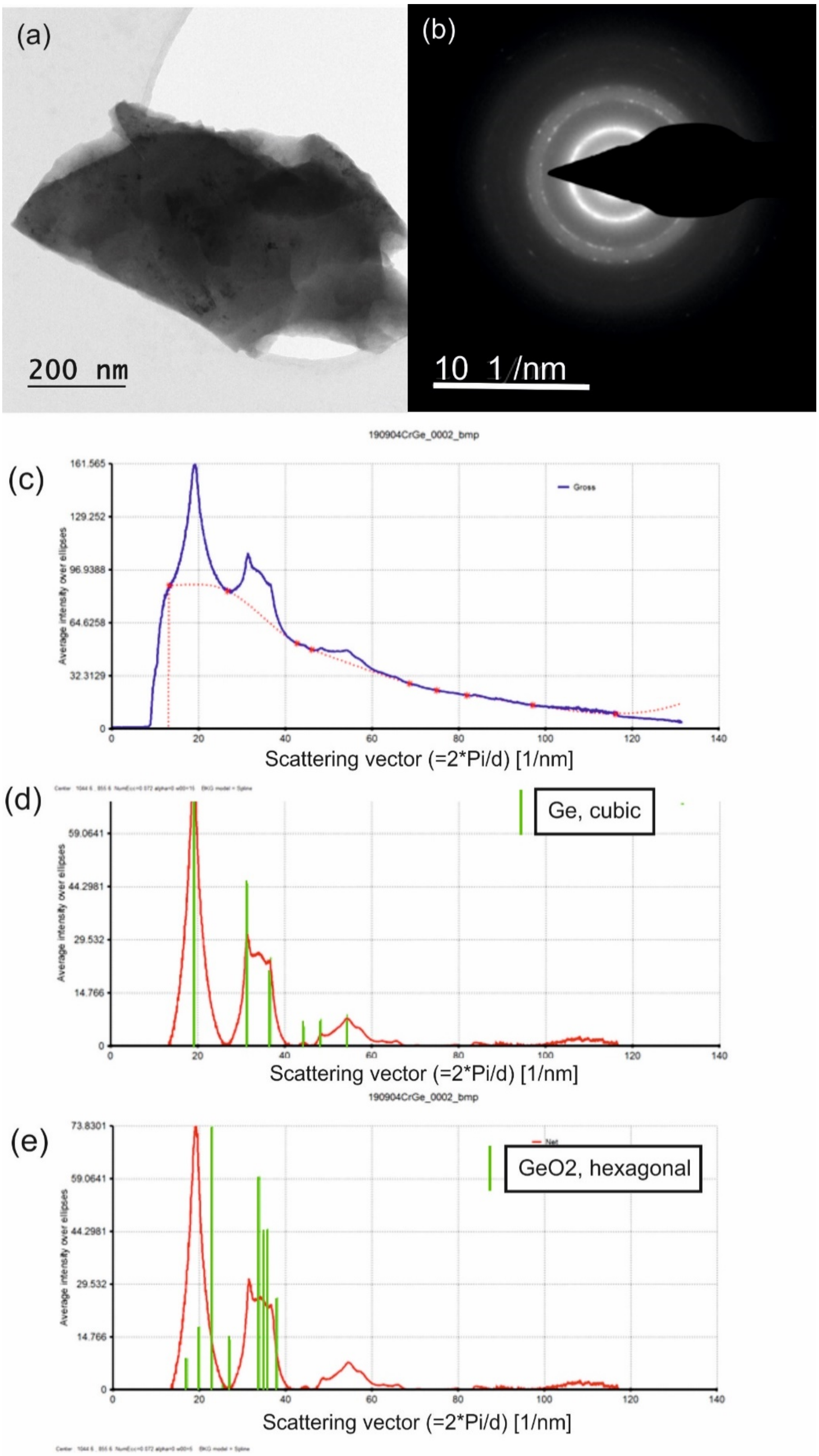

Figure S6: SAED analysis of an irregular object. (a) SEM photo, (b) SAED image, (c) SAED image converted into an intensity diffractogram, before background substraction, and (d) Ge cubic (PDF 89-3833) and (e) Ge hexagonal (PDF 51-0767) markers in diffractogram after background substraction. Diffuse rings and distinct points indicate an amorphous phase and the cubic/hexagonal phase, respectively.

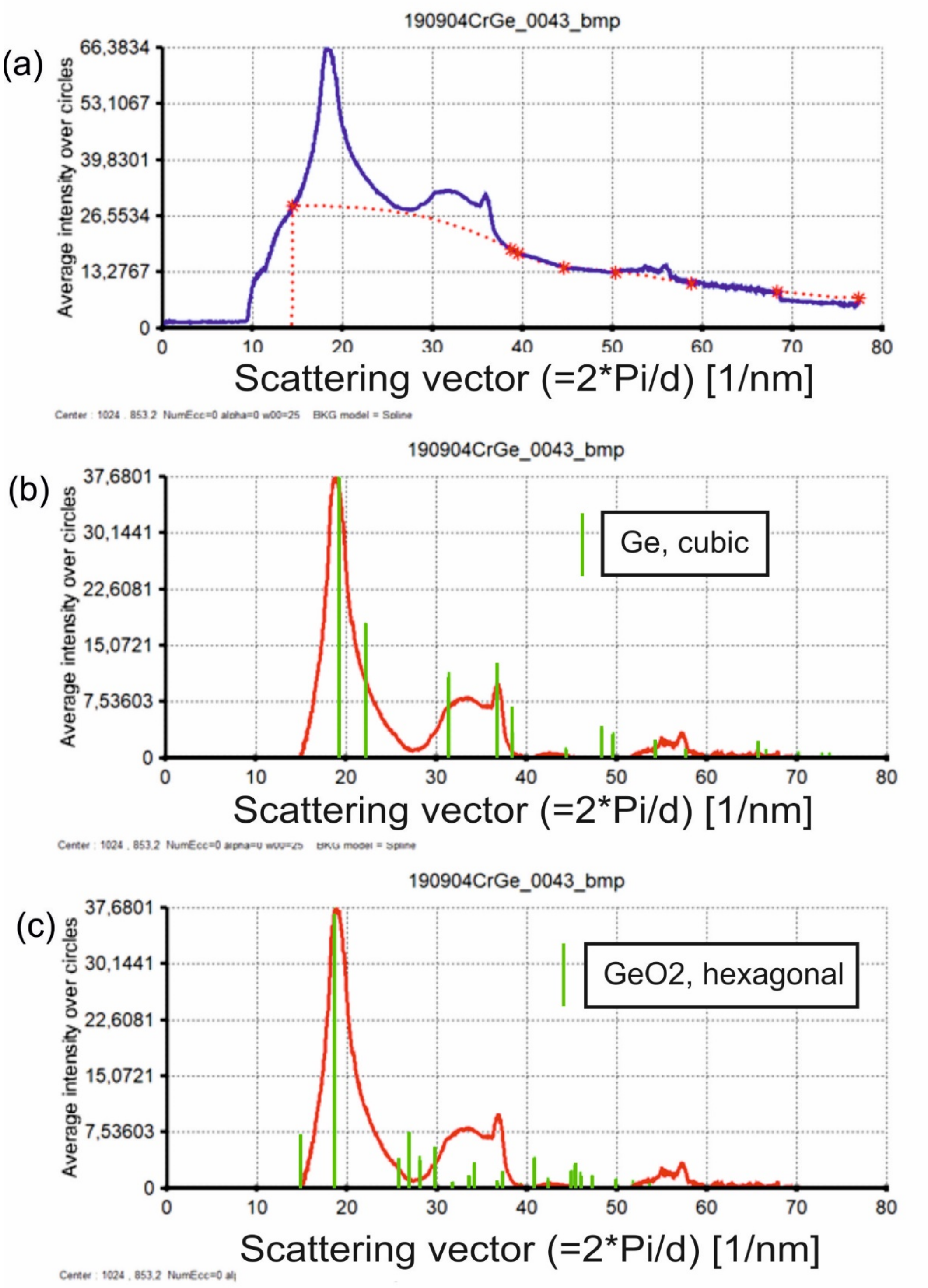

Figure S7: SAED image (Figure 3b) converted into (a) an intensity diffractogram, before background substraction, (b) Ge cubic (PDF 65-9202) and (c) GeO_2 hexagonal (PDF 83-0544) markers in diffractogram after background substraction.

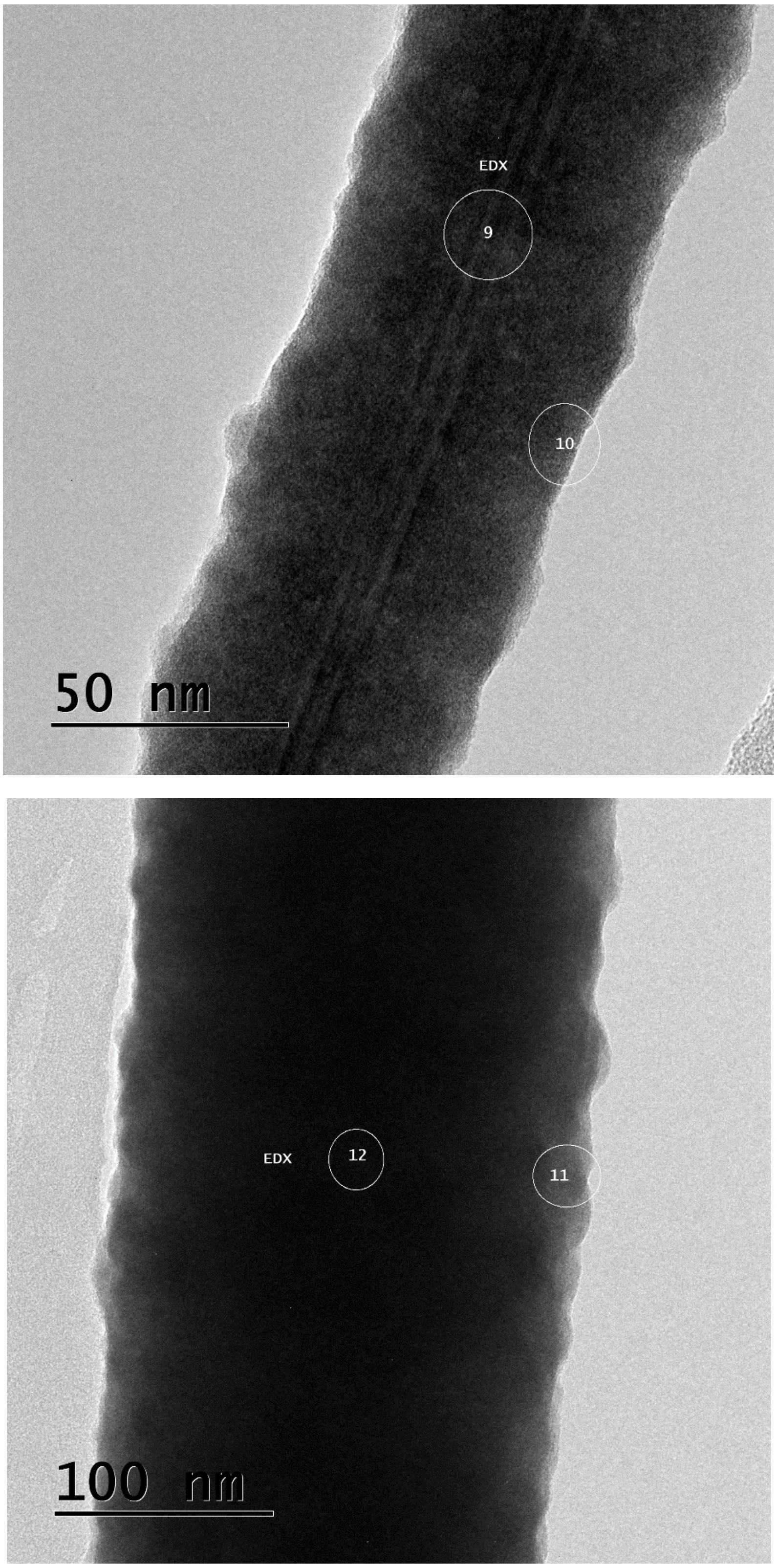

Figure S8: HRTEM images of a nanowire body with indicated EDX spots.

Elemental composition: [Cr]/[Ge]/[O]= 1:9.7:3.8, point 9- middle of NW comprising core+coating
[Cr]/[Ge]/[O]= 1:6.5:4.0, point 10 – only NW coating
[Cr]/[Ge]/[O]= 1:7.0:4.3, point 11 - only NW coating
[Cr]/[Ge]/[O]= 1:9.0:4.0, point 12 - middle of NW comprising core+coating

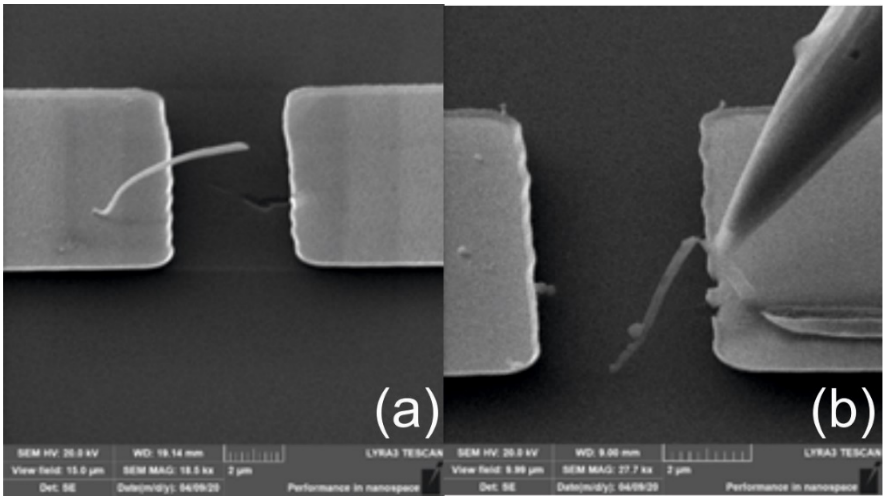

Figure S9: (a, b) unsuccessful attempt to transfer the NW onto lithographic pads.

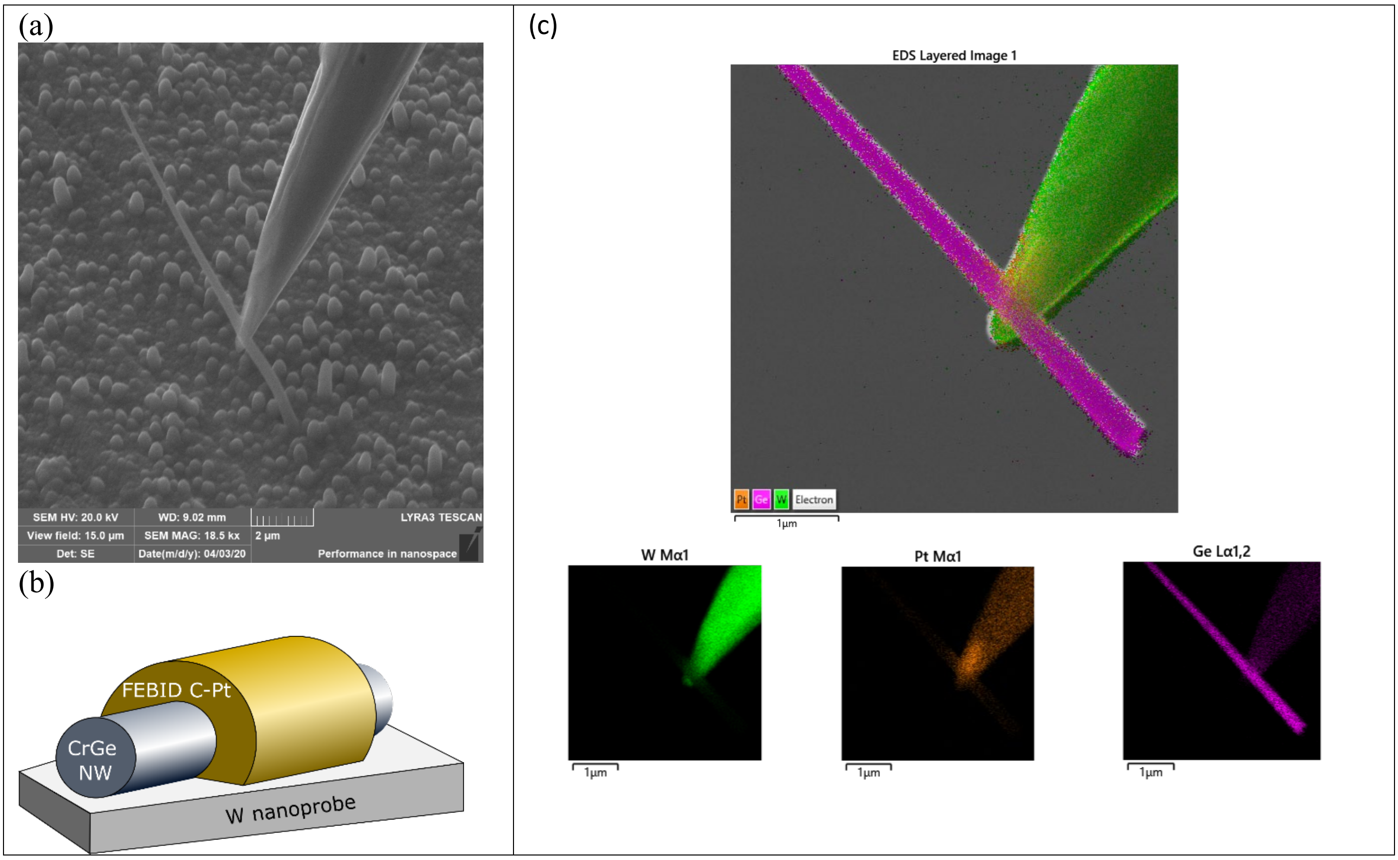

Figure S10: (a) SEM image of the GeCr nanowire that was contacted by the nanomanipulator tip for *I*–*V* measurements, (b) the detail of the point contact between the $CrGe_x$ nanowire and the nanomanipulator tip, and (c) elemental mapping of the point contact between the $CrGe_x$ nanowire and tungsten tip.

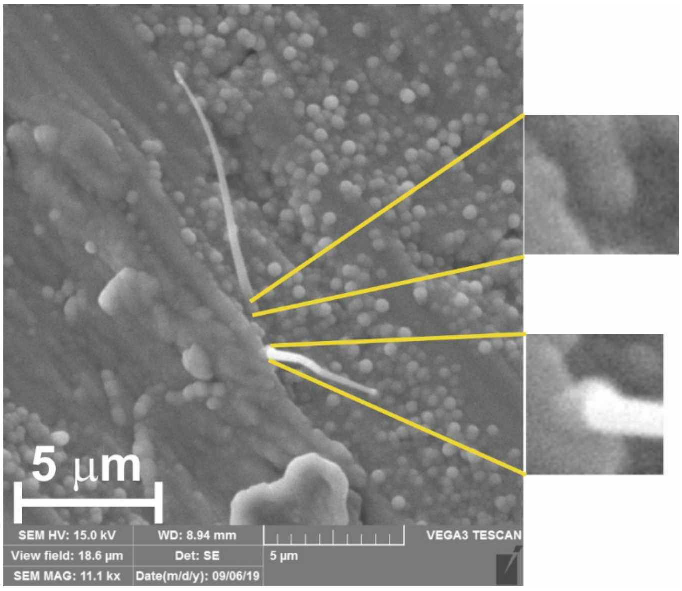

Figure S11: Typical nanowire bottoms corresponding to original nanoballs from which the nanowires started to grow.

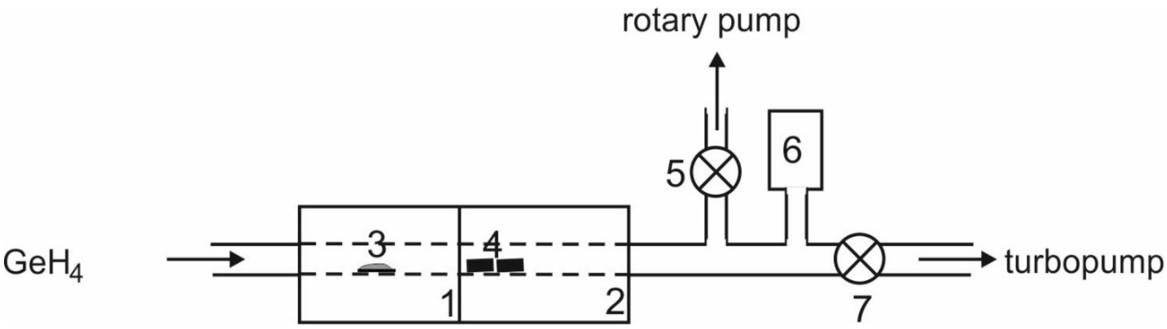

Figure S12: CVD set-up; 1,2 - twin furnace, first (max. 500 °C) and second part (max. 1200 °C), 3 – substrate with $Cr(acac)_3$, 4 – substrates for deposits, 5 – valve, 6 – pressure gauge, 7 – gate valve.
